# Repeated Electroacupuncture Persistently Elevates Adenosine and Ameliorates Collagen-Induced Arthritis in Rats

**DOI:** 10.1155/2016/3632168

**Published:** 2016-01-31

**Authors:** Tian-shen Ye, Zhong-heng Du, Zhi-hui Li, Wen-xia Xie, Ka-te Huang, Yong Chen, Zhou-yang Chen, Huan Hu, Jun-lu Wang, Jian-Qiao Fang

**Affiliations:** ^1^Department of Acupuncture, The First Affiliated Hospital of Wenzhou Medical University, Wenzhou, Zhejiang 325000, China; ^2^Chengdu Integrated Traditional Chinese and Western Medicine Hospital, Chengdu, Sichuan 610000, China; ^3^Department of Pathology, The First Affiliated Hospital of Wenzhou Medical University, Wenzhou, Zhejiang 325000, China; ^4^Department of Anesthesia, The First Affiliated Hospital of Wenzhou Medical University, Wenzhou, Zhejiang 325000, China; ^5^Department of Neurobiology and Acupuncture Research, The Third Clinical Medical College, Zhejiang Chinese Medical University, Hangzhou, Zhejiang 310053, China

## Abstract

The aim of this paper was to investigate the effect of repeated electroacupuncture (EA) over 21 days on the adenosine concentration in peripheral blood of rats with collagen-induced arthritis (CIA). Wistar rats were divided into three groups of 6 animals each: sham-control, CIA-control, and CIA-EA. We determined the adenosine concentration in peripheral blood and assessed pathological changes of ankle joints. Quantitative reverse-transcription-polymerase chain reaction was used to determine mRNA levels of ecto-5′-nucleotidase (CD73), adenosine deaminase (ADA), and tumor necrosis factor-alpha (TNF-*α*). Immunohistochemical staining was used to detect expression of ADA and CD73 in synovial tissue. Repeated EA treatment on CIA resulted in the persistence of high concentrations of adenosine in peripheral blood, significantly reduced pathological scores, TNF-*α* mRNA concentrations, and synovial hyperplasia. Importantly, EA treatment led to a significant increase in CD73 mRNA levels in peripheral blood but was associated with a decrease of CD73 immunostaining in synovial tissue. In addition, EA treatment resulted in a significant decrease of both ADA mRNA levels in peripheral blood and ADA immunostaining in synovial tissue. Thus, repeated EA treatment exerts an anti-inflammatory and immunoregulatory effect on CIA by increasing the concentration of adenosine. The mechanism of EA action may involve the modulation of CD73 and ADA expression levels.

## 1. Introduction

Rheumatoid arthritis (RA) is a systemic autoimmune disease that primarily consists of chronic and symmetric polyarthritis. Its main pathology is characterized by synovial hyperplasia associated with damage to cartilage and bone. Tumor necrosis factor-*α* (TNF-*α*), interleukin-1 (IL-1), and interleukin-6 (IL-6) expression levels are increased in serum of RA patients or experimental animals with collagen-induced arthritis (CIA) [1, 2]. The strong expression of TNF-*α* in synovial tissue of RA patients has been demonstrated by the study of Li [3]. TNF-*α* stimulates synovial cells to produce proinflammatory agents such as NO, IL-1, and collagenase, which destroys surrounding cells and tissues. In addition, proinflammatory agents activate the proliferation and differentiation of synovial fibroblasts [4, 5]. In a study carried out by Ouyang et al. [5], the investigators found that TNF-*α* expression levels in blood and synovia of patients with RA significantly decreased after EA treatment, compared to controls. In this study, needles were inserted in EA and control patients in an identical fashion, but control patients did not receive electrostimulation. Ouyang et al. [6] also reported that EA effectively decreased expression levels of the proinflammatory cytokines IL-1 and IL-6 and increased expression levels of the anti-inflammatory cytokines IL-4 and IL-10 in peripheral blood and synovial fluid from patients with RA. Other studies [7, 8] have also demonstrated that EA treatment using the Stomach 36 (ST36) acupoint increased the threshold of pain and reduced swelling in RA patients, as well as the expression levels of TNF-*α*, in both serum and synovial fluid. This was associated with reduced synovial hyperplasia and cartilage damage, which resulted in delayed ankylosis of the affected joints.

Recently, there has been increasing focus on the role of adenosine in RA. Adenosine is an important messenger in the anti-inflammatory auto- or paracrine signal transduction pathway and is widely distributed among various organs and tissues. Ottonello et al. [9] characterized anti-inflammatory activity in synovial fluid from patients with RA as being dependent on the presence of adenosine. In the absence of pathologic conditions, the extracellular concentration of adenosine is very low. However, under conditions of hypoxia, inflammation, or infection the extracellular adenosine concentration increases rapidly and plays an anti-inflammatory and immunoregulatory role. Moreover, the concentration of adenosine in synovial fluid was closely related to the inhibition of neutrophil apoptosis. Goldman et al. [10] determined that the concentration of adenosine in close proximity to the ST36 acupoint significantly increased after acupuncture was performed using this point.

In addition, Yim et al. [11] reported that acupuncture using ST36 in mice with CIA reduced joint damage, inhibited proinflammatory cytokines IL-6, TNF-*α*, and interferon-*γ* serum levels, and affected immune cells including CD69+/CD3e+ cells and CD11a+/CD19+ cells, to modulate immune functions. Previous studies from our group [12] demonstrated that EA using acupoints ST36 and Spleen 6 (SP6) in a murine CIA model similarly reduced joint damage, inhibited proinflammatory cytokine TNF-*α* expression, and exerted anti-inflammatory effects through the local elevation of adenosine and subsequent signaling through A_2A_R. However, we also found that EA did not increase the expression of A_2A_R. Based on our previous findings, we realized that the anti-inflammatory effect of EA on rats with CIA is associated with elevated adenosine levels, and we hypothesized that adenosine levels need to remain high after EA in order to be effective. In order to achieve persistent and high adenosine levels after EA, we hypothesize that EA affects the activity of two key enzymes that affect adenosine metabolism: ecto-5′-nucleotidase (CD73) and adenosine deaminase (ADA). CD73 dephosphorylates adenosine monophosphate (AMP), thereby generating adenosine, while ADA deaminates and catabolizes adenosine to inosine [13], which effectively decreases adenosine levels. Together, the activities of ADA and CD73 determine the concentration of adenosine.

## 2. Materials and Methods

### 2.1. Animals

Eighteen male Wistar rats (4-5 weeks of age, weighing 120 ± 10 g) were used for this study. Rats were purchased from the Shanghai Silaike Laboratory Animal Co., Ltd. (Shanghai, China), housed in a controlled environment, and provided with standard rodent chow and water. Animal care and experimental procedures were performed according to the Regulations on the Care and Use of Experimental Animals. Six rats were randomized into the sham-control group, while the remaining rats were subjected to the induction of CIA.

### 2.2. Induction of CIA

Type II collagen extracted from bovine articular cartilage (CII, Chondrex, USA) was dissolved in 0.05 M of acetic acid (2 mg/mL) at 4°C overnight. Then, the solution was emulsified in an equal volume of complete Freund's adjuvant (CFA, Chondrex, USA). Rats were injected intradermally at the base of the tail with 0.5 mL of the emulsion. At day 21 after the first injection, rats received a second injection with the emulsion as described before at the base of the tail close to the previous injection site.

### 2.3. Clinical Assessment of CIA

Fifteen days after the primary immunization, the incidence of arthritis was determined based on the number of arthritic joints, and the arthritic index with a range of 0 to 4 was determined (score: 0 = normal; 1 = swelling of at least one toe joint; 2 = swelling of paws; 3 = swelling of all foot paws, but not involving the ankle joints; 4 = swelling of all foot paws involving the ankle joints). The arthritic index for each rat was calculated by adding up the four scores of individual paws, allowing a maximum score of 16 per rat. CIA was regarded as induced when the score was higher than one in more than two joints or higher than two in more than one joint. Mice with a score ≥4 were included in any of the experimental groups.

### 2.4. Experimental Groups

Rats were divided into the CIA-control, CIA-EA, and sham-control groups. At 15 days after the first immunization, 12 rats with an arthritis score >4 were chosen and randomly divided into the CIA-control group and CIA-EA group (*n* = 6, each group). All rats in these two groups received a booster injection of CII in CFA emulsion at the base of the tail on day 21 after the primary immunization, in order to further enhance the immune response. Rats in the sham-control group were injected with 100 *μ*L of 0.01 M acetic acid instead using the same route and were not subjected to any treatment other than immobilization.

### 2.5. EA Treatment

Rats were immobilized during EA treatment using the gentle fixation method described previously [12]. Compared with common rigid restrainers, this method allows rats to have more freedom to move their body and limbs, provides a dark and warm environment, and minimizes stress during the experimental treatment. All groups were immobilized for 30 minutes a day during the treatment period. Sterilized disposable stainless steel acupuncture needles (0.25 × 30 mm; Hua Tuo Acupuncture, China) were used in this study. The ST36 and SP6 acupoints of rats were determined according to Chinese Acupuncture and Moxibustion [11, 14]. ST36 is located longitudinally three body inches below the knee joint, transversely in the middle of the tibialis anterior muscle, while SP6 is located three body inches above the apex of the medial malleolus, behind the tibia. Needles were inserted into the ST36 acupoint up to a depth of approximately 7 mm, while needles were inserted into the SP6 acupoint up to a depth of 5 mm. Thereafter, rats were electrically stimulated with a continuous rectangular wave current (2 Hz) for 30 minutes every 24 hours starting from day 15 to day 36 after the primary immunization (*n* = 6). The voltage (2.0–2.5 mV) was adjusted until local muscle contractions were seen. EA treatment was administered to the left and right hind legs, simultaneously.

### 2.6. High Performance Liquid Chromatography (HPLC)

#### 2.6.1. Preparation of Test Plasma

On day 37 (one day after the last EA treatment), rats were anesthetized by intraperitoneal administration of 10% chloral hydrate. Then, 2 mL of blood was collected from the plexus orbitalis and transferred immediately into heparinized tubes. One mL of blood was used for plasma preparation as follows. Plasma was collected after centrifugation at 900 g for one minute. Then, 300 *μ*L of plasma was mixed with 300 *μ*L of 0.4 M perchloric acid to precipitate all proteins.

After centrifugation at 11,000 g for 20 minutes, the supernatant was collected and adjusted to a pH of approximately 7.4 by addition of 1 mM KOH. After another centrifugation at 11,000 g for 20 minutes, the supernatant was collected and the extracellular concentration of adenosine was analyzed by HPLC.

#### 2.6.2. HPLC

The HPLC column (4.6 × 250 mm; Yi Lite, Dalian, China) was filled with Hypersil ODS 5 *μ*m. Methanol was used as mobile phase A. An equimolar mixture of 50 mM of Na_2_HPO_4_ and 50 mM of NaH_2_PO_4_ was used as mobile phase B. The mobile phase was a mixture of mobile phases A and B at a constant ratio of 20 : 80. HPLC was run at a speed of 1 mL/min at 25°C. Sample volume was 20 *μ*L. Adenosine was detected using a UV detector measuring the absorbance at 254 nm.

### 2.7. Histological Examination (HE)

Ankle joints were removed from every mouse and fixed in 4% paraformaldehyde solution for 24 hours. Ankle joints were decalcified in 10% EDTA (pH 8.0; Sigma, USA) for 2-3 weeks and embedded in paraffin. Paraffin-embedded joints were sectioned (4 *μ*m sections), deparaffinized, and stained with hematoxylin and eosin for histological assessment. Arthritis severity in histologic samples was determined by cumulative assessment of synovial inflammation. Arthritis damage (histological damage score) was evaluated and scored by an investigator blinded to the treatment regimen. The following morphological criteria were applied: score 0, no damage; score 1, edema; score 2, presence of inflammatory cells; score 3, bone resorption. Each section was examined at 40x magnification.

### 2.8. Quantitative Reverse-Transcription-PCR (qRT-PCR)

#### 2.8.1. Single Specimen Nuclear Cell Preparation

The remaining 1 mL of blood was diluted to 2 mL with phosphate-buffered saline (PBS). 2 mL of lymphocyte separation medium (LTS1083, TBD) was prepared in a graduated centrifuge tube. With the tube fixed at a 45° angle and using a capillary burette, the diluted heparinized blood was carefully overlaid onto the surface of the lymphocyte separation medium along the tube wall without disturbing the interface at 18–20°C.

After centrifugation for 20 minutes at 220 g, a capillary was gently inserted into the zone of turbidity; and along the wall of the tube, the layer of cells was gently aspirated and transferred into another centrifuge tube. After washing the cells three times with PBS, the supernatant was discarded and the cell pellet was stored at −80°C.

#### 2.8.2. qRT-PCR

Total RNA of the mononuclear cells was extracted using TRIzol reagent (Ambion, USA; 15596-026) according to manufacturer's instructions. RNA was resuspended in DEPC water and quantified by determining absorbance at 260 nm, while purity of the RNA was characterized by determining the ratio of absorbance at *A*260/280 nm with a UV spectrophotometer. Samples with absorbance ratios within 1.8–2.0 were selected for qRT-PCR. One *μ*g of RNA was reverse-transcribed to cDNA by a ReverTra Ace qPCR RT Kit (FSQ-101; Toyobo, Japan) according to manufacturer's instructions. TNF-*α*, CD73, and ADA mRNA levels were detected by SYBR Green Real-Time PCR Master Mix-Plus (QPK-212; Toyobo, Japan) according to manufacturer's instructions. Real-time PCR plates were run on an ABI-7500 Fast Real-Time PCR System with ABI Prism SDS 1.2 software (Applied Biosystems) for evaluation. Rat primer sequences for qPCR were as follows:

TNF-*α*: forward primer 5′-3′ TAGAAAGGGAATTGTGGCTCTG, reverse primer 3′-5′ TACTTCAGCGTCTCGTGTGTTT;

CD73: forward primer 5′-3′ GGTGGAATCCATGTGGTGTA, reverse primer 3′-5′ CATAGATGGGCACTCGACAC;

ADA: forward primer 5′-3′ GGTGCGAGAGGCTGTGGAC, reverse primer 3′-5′ CGAGTTGAGGGAGTAGTTGGC.

### 2.9. Immunohistochemistry (IHC) for Tissue Sections

Each paraffin-embedded block of ankle tissue was cut into 4 *μ*m sections, and the sections were transferred onto silanized glass slides for IHC staining. Tissue sections were deparaffinized and rehydrated through graded alcohols. Endogenous peroxidase was quenched by 10-minute incubation with 0.3% hydrogen peroxide. The sections were permeabilized with 0.1% Triton X-100 in PBS for 20 minutes and were incubated overnight at 4°C with rabbit monoclonal anti-ADA antibody (1 : 400 dilution, Abcam, ab175310) or rabbit monoclonal anti-CD73 antibody (1 : 300 dilution, Abcam, ab175396) at a dilution of 1 : 200. The slides were washed with PBS and incubated with horseradish peroxidase- (HRP-) labeled anti-rabbit immunoglobulin as the secondary antibody. Peroxidase activity was visualized by treating the slides with 3,3′-diaminobenzidine (DAB). Then, the slides were counterstained with hematoxylin. Each section was examined at high magnification (×400) and tested five times. Results were expressed as mean ± SD for six mice in each group. Human HeLa (ADA) and colorectal cancer (CD73) tissues were used as positive controls. A negative control was obtained by replacing the primary antibody with PBS.

### 2.10. Statistical Analysis

Values were expressed as mean ± SD. Comparison of means was carried out by ANOVA. All statistical analyses were performed with SPSS software version 19.0. *P* values <0.05 were considered statistically significant.

## 3. Results

### 3.1. Concentration of Adenosine in Peripheral Blood

**Figure 1:**
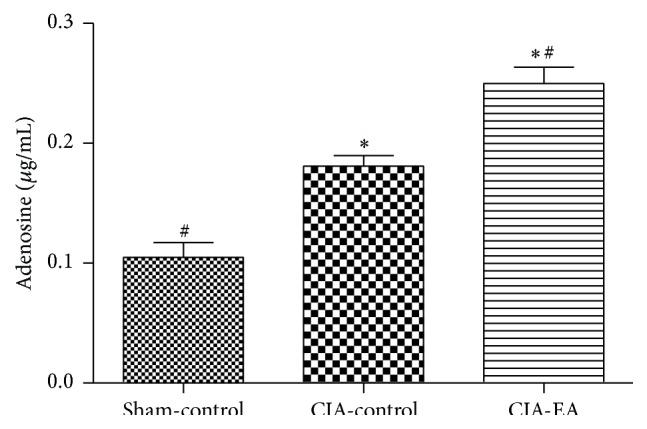
Effect of CIA and EA treatment on adenosine concentration in peripheral blood. Adenosine concentration in peripheral blood was determined by HPLC. Adenosine concentrations are expressed as mean ± SD. ^*∗*^ *P* < 0.01 versus the sham-control group. ^#^ *P* < 0.01 versus the CIA-control group.

Adenosine concentrations in peripheral blood of rats in the CIA-control and CIA-EA groups increased compared to the sham-control group (*P* < 0.01). More importantly, adenosine concentrations in the CIA-EA group significantly increased compared to the CIA-control group (*P* < 0.01; Figure 1, Table 1).

### 3.2. Histological Evaluation

**Figure 2:**
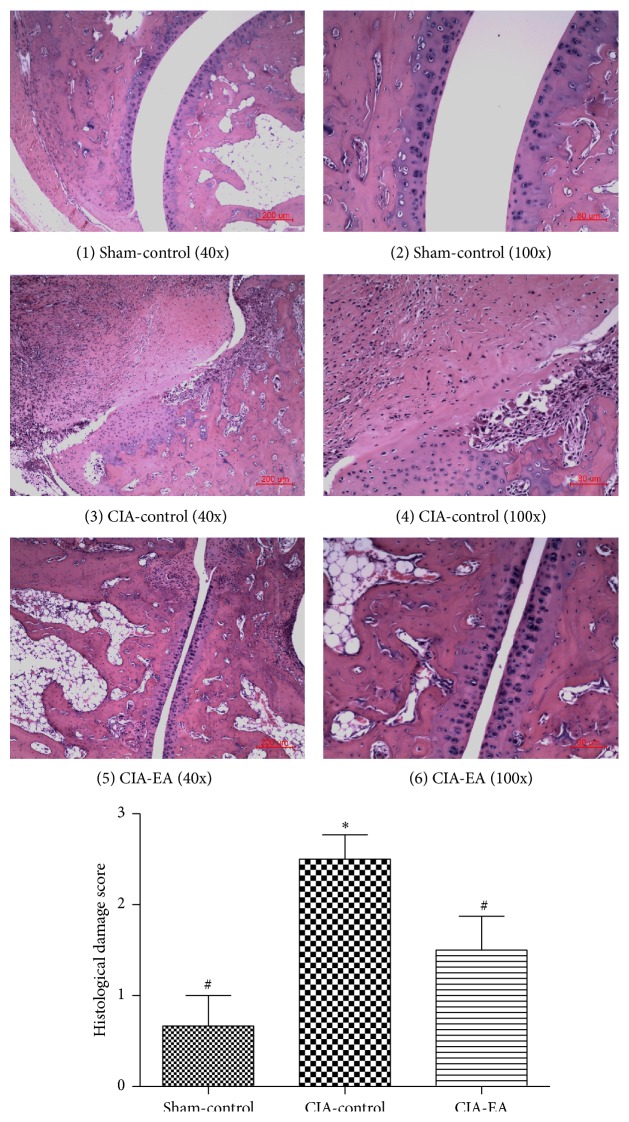
Effect of CIA and EA treatment on ankle joints. A normal ankle joint is shown in (1)-(2). Signs of severe arthritis with inflammatory cell infiltration, synovial hyperplasia, joint fusion, and bone erosion are depicted in (3)-(4). EA treatment of CIA joints that resulted in the alleviation of inflammation is revealed in (5)-(6). Data are expressed as mean ± SD. ^*∗*^ *P* < 0.01 compared with the sham-control group. ^#^ *P* < 0.05 compared with the CIA-control group.

Histological evaluation of ankle joints from mice in the CIA-control group revealed signs of severe arthritis with infiltration of inflammatory cells, synovial hyperplasia, and joint fusion, compared to mice in the sham-control group, which exhibited normal histology. Apparent histological alterations in joints of mice in the CIA-EA group appeared significantly ameliorated compared to mice in the CIA-control group (Figure 2, Table 2).

### 3.3. Effect of EA on TNF-*α* mRNA Expression in CIA

**Figure 3:**
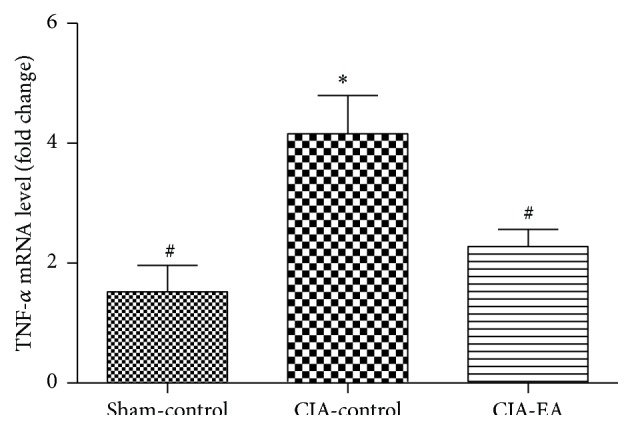
Effect of CIA and EA treatment on TNF-*α* mRNA levels in peripheral blood mononuclear cells. mRNA was determined by qRT-PCR. Data (fold change) are expressed as mean ± SD. ^*∗*^ *P* < 0.01 compared with the sham-control group. ^#^ *P* < 0.05 compared with the CIA-control group.

TNF-*α* mRNA expression in peripheral blood mononuclear cells significantly increased in the CIA-control group compared with the sham-control group (*P* < 0.01). Importantly, TNF-*α* mRNA expression levels decreased in the CIA-EA group compared with the CIA-control group (*P* < 0.05; Figure 3, Table 3).

### 3.4. Effect of EA on CD73 and ADA mRNA Expression in CIA

**Figure 4:**
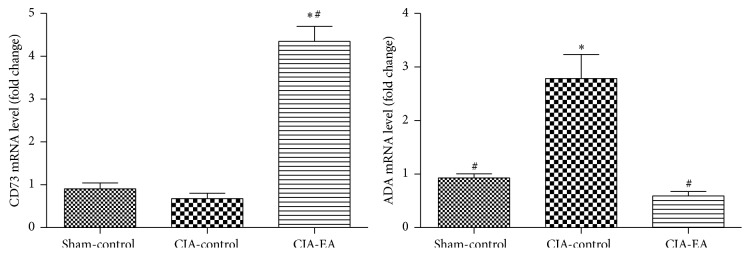
Effect of CIA and EA treatment on CD73 and ADA mRNA expression in peripheral blood mononuclear cells. mRNA was quantified by qRT-PCR. Data (fold change) are expressed as mean ± SD. ^*∗*^ *P* < 0.01 versus the sham-control group. ^#^ *P* < 0.01 versus the CIA-control group.

On the day after the last EA treatment, CD73 and ADA mRNA levels in peripheral blood mononuclear cells were quantified by qRT-PCR. We found that the mRNA expression of CD73 was significantly elevated in the CIA-EA group compared with the CIA-control group (*P* < 0.01). However, there was no significant difference in CD73 mRNA expression between the sham-control and CIA-control groups (*P* > 0.05). The mRNA expression of ADA was also significantly elevated in the CIA-control group, compared with the sham-control group (*P* < 0.01). Interestingly, ADA mRNA expression was significantly lower in the CIA-EA group, compared to the CIA-control group (*P* < 0.01). There was no significant difference in ADA mRNA expression between the sham-control and CIA-EA groups (*P* > 0.05; Figure 4, Tables 4 and 5).

### 3.5. CD73 and ADA Protein Expression in Synovial Tissue

**Figure 5:**
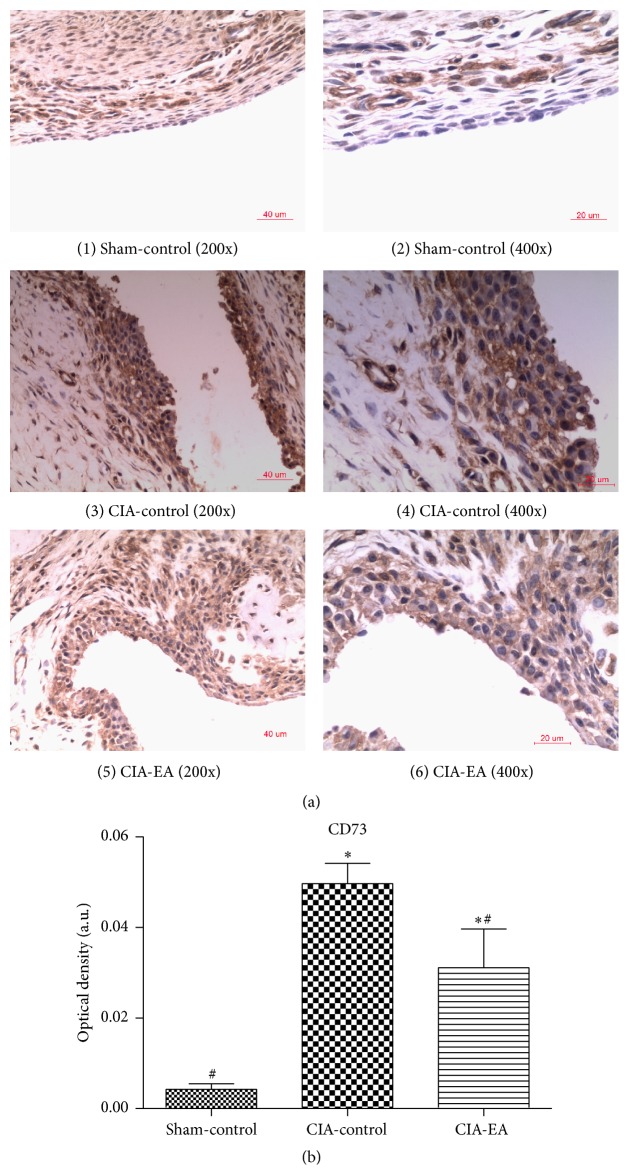
Effect of CIA and EA treatment on CD73 expression in synovial membranes. (a) (1)-(2), (3)-(4), and (5)-(6) show the immunostaining of CD73 in synovial membranes in the sham-control, CIA-control, and CIA-EA groups, respectively. (b) Quantification of the immunostaining of sections of the synovial membrane. Each section was examined at high magnification. Results represent the mean of five different fields on each section. Data are expressed as mean ± SD. ^*∗*^ *P* < 0.01 versus the sham-control group. ^#^ *P* < 0.05 versus the CIA-control group. Magnification: ×200 ((1), (3), and (5)), ×400 ((2), (4), and (6)).

First, immunohistochemical analysis of synovial tissue from ankle joints revealed that the immunostaining of CD73 was stronger in rats in the CIA-control group compared to the sham-control group (*P* < 0.01). Immunostaining of CD73 was weaker in synovial tissue of rats in the CIA-EA group compared to the CIA-control group (*P* < 0.05) but was stronger compared to rats in the sham-control group (*P* < 0.05; Figure 5, Table 6).

**Figure 6:**
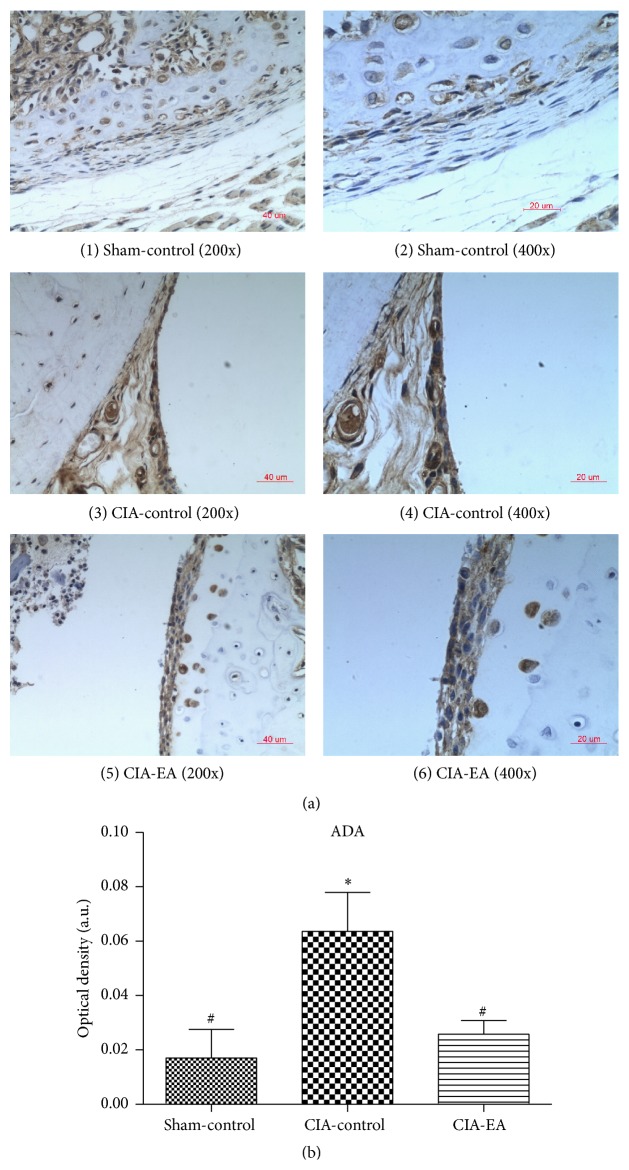
Effect of CIA and EA treatment on ADA expression in synovial membranes. (a) (1)-(2), (3)-(4), and (5)-(6) show the immunostaining of ADA in synovial membranes in the sham-control, CIA-control, and CIA-EA groups, respectively. (b) Quantification of the immunostaining of sections of the synovial membrane. Each section was examined at high magnification. Results represent the mean of five different fields on each section. Data are expressed as mean ± SD. ^*∗*^ *P* < 0.01 versus the sham-control group; ^#^ *P* < 0.05 versus the CIA-control group. Magnification: ×100 (3), ×200 ((1) and (5)), and ×400 ((2), (4), and (6)).

Second, immunostaining of ADA was much stronger in rats in the CIA-control group compared to rats in the sham-control group (*P* < 0.01). ADA protein expression significantly decreased in rats in the CIA-EA group compared to rats in the CIA-control group (*P* < 0.05; Figure 6, Table 7).

## 4. Discussion

Goldman et al. [10] found that acupuncture resulted in a significant increase in adenosine near the acupuncture site, which may have been related to sympathetic nerve stimulation by acupuncture. Local adenosine concentrations returned to normal levels within 60 minutes. There are several known mechanisms that modulate local adenosine levels, and the most important mechanisms are the activities of CD73 and ADA. One mechanism to account for the increase in extracellular concentrations of adenosine is related to the increase in CD73 activity. Hydrolysis of AMP by CD73 occurs in the extracellular compartment, resulting in the generation of extracellular adenosine, which is irreversible [15]. Oliveira et al. [16] reported that the CD73 pathway is responsible for the increased production of extracellular adenosine by Treg cells, which in turn exerts an immunosuppressive action on activated T cells via A_2A_R activation. Similarly, Chrobak et al. [17] suggested that CD73 deficiency resulted in increased production of proinflammatory cytokines in joints, increased Th1 T cell responses, and increased joint destruction in CD73-deficient CIA mice, consistently indicating an anti-inflammatory role of CD73. Similarly, Szabo and Pacher [18] suggested that CD73 plays an important role in the extracellular conversion of AMP to adenosine and thereby acts as a checkpoint that determines whether the extracellular environment is proinflammatory or anti-inflammatory.

In this study, we found that EA treatment was associated with a lower degree of inflammation on the histological evaluation of ankle joints (less inflammatory cell infiltration, synovial hyperplasia, and joint fusion) and reduced levels of TNF-*α* mRNA in peripheral blood mononuclear cells compared to CIA-control rats. However, the analysis of CD73 expression in affected synovial tissue from the ankle joints of rats in the CIA-control group exhibited strong local immunostaining, while EA treatment resulted in decreased immunostaining. This means that EA was associated with both decrease in inflammation and decrease in CD73 expression, limiting the potential role of local CD73 expression for the anti-inflammatory effect of EA treatment. More interestingly, EA treatment significantly increased CD73 mRNA expression in peripheral blood mononuclear cells in rats with CIA, suggesting a strong systemic effect of EA. This observed association between the increased expression of CD73 in blood mononuclear cells and decreased inflammation in CIA joints after EA suggests a role for the systemic effect of EA in the regulation of local inflammation. This seems to reflect the character of RA and CIA as a systemic disease that mostly manifests locally as joint inflammation.

Savic et al. [19] found that high levels of TNF-*α* and IL-1 cause the upregulation of CD73 in synovial fluid. This suggests that CD73 expression in synovial tissue is mostly determined by local levels of proinflammatory cytokines, which is in good agreement with our data.

In summary, this suggests that most of the clinical effect of EA, such as the anti-inflammatory effect clearly visible in our experimental system, seems to be mediated by the more systemic effect of EA, which is detectable in blood mononuclear cells and through increased plasma adenosine concentrations. This effect may be mediated locally by infiltrating leukocytes, for example, in the synovial space rather than the synovial tissue, and by adenosine entering the synovial fluid from blood.

In contrast to CD73, ADA activity leads to the conversion of adenosine to inosine, thereby reducing the extracellular concentration of adenosine [20]. Hitoglou et al. [21] reported that ADA activity was closely related to disease progression in RA, and disease recurrence was associated with high levels and high activity of ADA. A clinical study by Zakeri et al. [22] found significantly increased levels of ADA in patients with RA compared to osteoarthritis patients. In addition, Wang et al. [23] found that ADA protein concentrations in the cerebral cortex significantly decreased at 60 minutes after EA in an ischemia/reperfusion model in SD rats, and levels returned to normal within 120 minutes. At the same time, they found that adenosine increased at 120 minutes after EA. Moreover, it was reported that using adenosine antagonists that are nonspecific for particular adenosine receptors, such as caffeine, could reverse the inhibitory effect of acupuncture on inflammation [7]. Zylka [24] found that the inhibition of ADA by deoxycoformycin generally prolonged the antinociceptive effects of acupuncture. All reported data on ADA are consistent and in agreement with the proinflammatory effect of ADA. Interestingly, the anti-inflammatory effects of EA seem to be similar to the effects of methotrexate. Both treatments result in an increase in the extracellular concentration of adenosine and the downregulation of ADA [25–27].

In our study, ADA significantly increased in both peripheral blood and synovial tissue from ankle joints of rats in the CIA-control group, compared to rats in the sham-control group. EA resulted in a decrease in local and peripheral ADA expression in CIA rats, consistent with the regulation of ADA expression levels by inflammatory factors. Therefore, the decrease in ADA expression may be a result of the combined action of an indirect mechanism of EA action via inflammation relief and a direct mechanism of the downregulation of ADA expression by EA. However, this question needs to be further clarified.

## 5. Conclusions and Outlook

**Figure 7:**
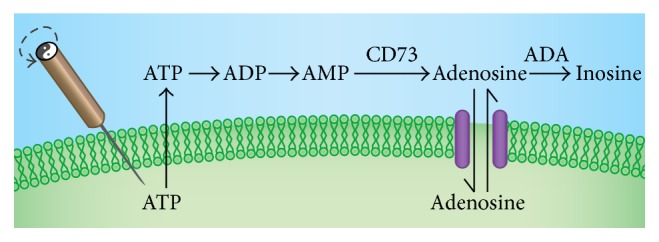
Metabolism of adenosine. Acupuncture causes ATP release. ATP is metabolized extracellularly to AMP by ectonucleotidases. CD73 hydrolyzes AMP to adenosine. Finally, adenosine is degraded to inosine by ADA or transported back into cells.

In summary, EA treatment for 21 days affects the activity of CD73 and ADA and leads to persistently elevated concentrations of adenosine in peripheral blood of rats with CIA. This was associated with a lower degree of inflammation on the histological evaluation of ankle joints and reduced TNF-*α* mRNA levels in peripheral blood mononuclear cells, compared to rats in the CIA-control group. This persistent increase in adenosine concentration may play an anti-inflammatory and joint damage reduced role through the binding and activation of adenosine receptors (Figure 7). Adenosine acts through four G-protein coupled receptors, and the A_2A_R pathway is particularly important among them. The contribution of other adenosine receptors remains to be studied.

## Figures and Tables

**Table 1:** Effect of EA treatment on adenosine concentrations in peripheral blood (*μ*g/mL).

Treatment group	*n*	Adenosine concentration
Sham-control	6	0.10 ± 0.03^#^
CIA-control	6	0.18 ± 0.02^*∗*^
CIA-EA	6	0.25 ± 0.03^*∗*,#^

^*∗*^ *P* < 0.01 versus the sham-control group; ^#^ *P* < 0.01 versus the CIA-control group.

**Table 2:** Effect of CIA and EA treatment on ankle joint histology.

Treatment group	*n*	Histological damage score
Sham-control	6	0.67 ± 0.82^#^
CIA-control	6	2.50 ± 0.66^*∗*^
CIA-EA	6	1.50 ± 0.91^#^

^*∗*^ *P* < 0.01 versus the sham-control group; ^#^ *P* < 0.05 versus the CIA-control group.

**Table 3:** Effect of EA treatment on TNF-*α* mRNA levels in peripheral blood mononuclear cells (fold change).

Treatment group	*n*	TNF-*α* mRNA
Sham-control	6	1.52 ± 1.08^#^
CIA-control	6	4.16 ± 1.57^*∗*^
CIA-EA	6	2.28 ± 0.70^#^

^*∗*^ *P* < 0.01 versus the sham-control group; ^#^ *P* < 0.05 versus the CIA-control group.

**Table 4:** Effect of EA treatment on CD73 mRNA expression in peripheral blood mononuclear cells (fold change).

Treatment group	*n*	CD73 mRNA
Sham-control	6	0.91 ± 0.33
CIA-control	6	0.68 ± 0.30
CIA-EA	6	4.35 ± 0.86^*∗*,#^

^*∗*^ *P* < 0.01 versus the sham-control group; ^#^ *P* < 0.01 versus the CIA-control group.

**Table 5:** Effect of EA treatment on ADA mRNA expression in peripheral blood mononuclear cells (fold change).

Treatment group	*n*	ADA mRNA
Sham-control	6	0.93 ± 0.19^#^
CIA-control	6	2.79 ± 1.10^*∗*^
CIA-EA	6	0.59 ± 0.20^#^

^*∗*^ *P* < 0.01 versus the sham-control group; ^#^ *P* < 0.01 versus CIA-control group.

**Table 6:** Effect of EA treatment on CD73 protein expression in synovial membranes (absorbance, arbitrary units).

Treatment group	*n*	CD73
Sham-control	6	0.004 ± 0.003^#^
CIA-control	6	0.050 ± 0.011^*∗*^
CIA-EA	6	0.031 ± 0.021^*∗*,#^

^*∗*^ *P* < 0.01 versus the sham-control group; ^#^ *P* < 0.05 versus the CIA-control group.

**Table 7:** Effect of EA treatment on ADA protein expression in synovial membranes (absorbance, arbitrary units).

Treatment group	*n*	ADA
Sham-control	6	0.017 ± 0.026^#^
CIA-control	6	0.064 ± 0.035^*∗*^
CIA-EA	6	0.026 ± 0.012^#^

^*∗*^ *P* < 0.01 versus the sham-control group; ^#^ *P* < 0.05 versus the CIA-control group.

## References

[1] Harris E. D. (1990). Rheumatoid arthritis. Pathophysiology and implications for therapy. *The New England Journal of Medicine*.

[2] van den Berg W. B. (2005). Animal models of arthritis. What have we learned?. *Journal of Rheumatology Supplement*.

[3] Li S.-F. (2007). Levels and significance of TNF-*α* in peripheral blood and synovial fluid form patients with rheumatoid arthritis. *European Journal of Medical Research*.

[4] Redich K. P., Hayen S., Ricct R. (2002). Ostecolasts are essential for INF-mediated joint destruction. *Arthritis Research*.

[5] Ouyang B.-S., Gao J., Che J.-L. (2011). Effect of electro-acupuncture on tumor necrosis factor-*α* and vascular endothelial growth factor in peripheral blood and joint synovia of patients with rheumatoid arthritis. *Chinese Journal of Integrative Medicine*.

[6] Ouyang B.-S., Che J.-L., Gao J. (2010). Effects of electroacupuncture and simple acupuncture on changes of IL-1, IL-4, IL-6 and IL-10 in peripheral blood and joint fluid in patients with rheumatoid arthritis. *Zhongguo Zhen Jiu*.

[7] Liu G.-Y., Li X.-P., Ye T.-S. (2012). Effect of acupuncture anti-inflammatory effects on adenosine receptor antagonist-caffeine in CIA rats. *Chinese Archives of Traditional Chinese Medicine*.

[8] Fang J.-Q., Shao X.-M., Ma G.-Z. (2009). Effect of electroacupuncture at ‘Zusanli’ (ST 36) and ‘Sanyinjiao’ (SP 6) on collagen-induced arthritis and secretory function of knee-joint synoviocytes in rats. *Acupuncture Research*.

[9] Ottonello L., Cutolo M., Frumento G. (2002). Synovial fluid from patients with rheumatoid arthritis inhibits neutrophil apoptosis: role of adenosine and proinflammatory cytokines. *Rheumatology*.

[10] Goldman N., Chen M., Fujita T. (2010). Adenosine A1 receptors mediate local anti-nociceptive effects of acupuncture. *Nature Neuroscience*.

[11] Yim Y.-K., Lee H., Hong K.-E. (2007). Electro-acupuncture at acupoint ST36 reduces inflammation and regulates immune activity in collagen-induced arthritic mice. *Evidence-Based Complementary and Alternative Medicine*.

[12] Li Q.-H., Xie W.-X., Li X.-P. (2015). Adenosine A2A receptors mediate anti-inflammatory effects of electroacupuncture on synovitis in mice with collagen-induced arthritis. *Evidence-Based Complementary and Alternative Medicine*.

[13] Haskó G., Cronstein B. N. (2004). Adenosine: an endogenous regulator of innate immunity. *Trends in Immunology*.

[14] Deng L., Gan Y., He S. (1997). *Chinese Acupuncture and Moxibustion*.

[15] Beavis P. A., Stagg J., Darcy P. K., Smyth M. J. (2012). CD73: a potent suppressor of antitumor immune responses. *Trends in Immunology*.

[16] Oliveira L., Correia A., Cristina Costa A. (2015). Deficits in endogenous adenosine formation by Ecto-5′-nucleotidase/CD73 impair neuromuscular transmission and immune competence in experimental autoimmune myasthenia gravis. *Mediators of Inflammation*.

[17] Chrobak P., Charlebois R., Rejtar P., El Bikai R., Allard B., Stagg J. (2015). CD73 plays a protective role in collagen-induced arthritis. *The Journal of Immunology*.

[18] Szabo C., Pacher P. (2012). The outsiders: emerging roles of ectonucleotidases in inflammation. *Science Translational Medicine*.

[19] Savic V., Stefanovic V., Ardaillou N., Ardaillou R. (1990). Induction of ecto-5′-nucleotidase of rat cultured mesangial cells by interleukin-1*β* and tumour necrosis factor-*α*. *Immunology*.

[20] Chan E. S. L., Fernandez P., Cronstein B. N. (2007). Adenosine in inflammatory joint diseases. *Purinergic Signalling*.

[21] Hitoglou S., Hatzistilianou M., Gougoustamou D., Athanassiadou F., Kotsis A., Catriu D. (2001). Adenosine deaminase activity and its isoenzyme pattern in patients with juvenile rheumatoid arthritis and systemic lupus erythematosus. *Clinical Rheumatology*.

[22] Zakeri Z., Izadi S., Niazi A. (2012). Comparison of adenosine deaminase levels in serum and synovial fluid between patients with rheumatoid arthritis and osteoarthritis. *International Journal of Clinical and Experimental Medicine*.

[23] Wang H.-F., Xia H.-L., Qin J.-L. (2013). The role of adenosine deaminase in the electroacupuncture preconditioning induced rapid tolerance to focal cerebral ischemia. *Chinese Journal of Integrative Medicine*.

[24] Zylka M. J. (2010). Needling adenosine receptors for pain relief. *Nature Neuroscience*.

[25] Cronstein B. N. (1996). Molecular therapeutics: methotrexate and its mechanism of action. *Arthritis and Rheumatism*.

[26] van Ede A. E., Laan R. F. J. M., De Abreu R. A., Stegeman A. B. J., van de Putte L. B. A. (2002). Purine enzymes in patients with rheumatoid arthritis treated with methotrexate. *Annals of the Rheumatic Diseases*.

[27] Salesi M., Ghazvini R. A., Farajzadegan Z. (2012). Serum adenosine deaminase in patients with rheumatoid arthritis treated with methotrexate. *Journal of Research in Pharmacy Practice*.

